# Retinal thickness is indicative of visual loss in patients with occipital lobe infarction

**DOI:** 10.3389/fneur.2025.1546439

**Published:** 2025-03-25

**Authors:** Xueling Bai, Le Cao, Hang Wang, William Robert Kwapong, Yuying Yan, Guina Liu, Junfeng Liu, Fayun Hu, Bo Wu

**Affiliations:** ^1^Department of Neurology, West China Hospital, Sichuan University, Chengdu, China; ^2^Department of Ophthalmology, West China Hospital, Sichuan University, Chengdu, China

**Keywords:** occipital lobe infarction, vision, retinal vessels, retinal structural thickness, infarct diameter

## Abstract

**Purpose:**

We explored the relationship between retinal thicknesses and vessels using optical coherence tomography angiography (OCT)/ OCT angiography (OCTA) and clinical outcomes in occipital lobe infarction (OI).

**Methods:**

A total of 52 OI patients and 105 controls underwent macular OCT/OCTA scans covering a 6 × 6 mm^2^ area around the fovea. The retinal nerve fiber layer (RNFL), ganglion cell-inner plexiform layer (GCIPL), superficial vascular complex (SVC), and deep vascular complex (DVC) were measured using the OCT/OCTA tool. All participants underwent a visual acuity examination.

**Results:**

OI patients showed reduced GCIPL thickness and lower SVC density but higher DVC density (all *p* < 0.001) compared to the controls, both in the whole area and across the four sectors. Eyes ipsilateral or contralateral to infarction showed reduced GCIPL thickness and lower SVC density (all *p* < 0.05). The GCIPL thickness was significantly correlated with the infarct diameter and visual acuity (both *p* < 0.05), while the SVC density was also significantly correlated with the infarct diameter (*p* = 0.002). The visual acuity showed a significant association with the infarct diameter (*p* < 0.001), and the reduction of the GCIPL partially mediated this effect (a proportion of the mediated effect at 15.17%, *p* = 0.028).

**Conclusion:**

GCIPL thinning may account for the effect of infarct diameter on visual acuity in OI patients. Future prospective studies are needed to assess OCT/OCTA as a potential marker of visual loss in OI.

## 1 Introduction

In occipital lobe infarction (OI), neurodegeneration plays a crucial role in its pathophysiology (1–3), showing a close association with visual abnormalities (4, 5). However, there remains a challenge in directly monitoring neuroaxonal loss *in vivo*.

In the retina, optical coherence tomography (OCT) allows for the visualization and quantification of the layers of neurons and axons in a non-invasive manner, with high inter-rater and intra-rater reproducibility. Clinically evident or sub-clinical optic nerve degeneration and retrograde degeneration caused by tissue damage in the occipital lobe (part of the posterior visual pathway) are frequent causes of OCT alterations in OI patients (2, 3, 6).

The peripapillary retinal nerve fiber layer (pRNFL), ganglion cell-inner plexiform layer (GCIPL), and ganglion cell complex (GCC) are the most sensitive and robust OCT metrics for detecting OI-associated neurodegeneration (7–10). Interestingly, OI patients may experience visual changes as a result of alterations in these retinal structures. Despite this, retinal metrics have not been studied in depth, and there is insufficient evidence regarding the association between cerebral imaging parameters and retinal metrics in OI patients.

The ability to use OCT as a marker of neurodegeneration in OI can be strengthened by understanding the extent to which OCT metrics are sensitive to OI. In this study, we aimed to achieve the following:

1. Explore retinal microvascular (with OCT angiography, OCTA) and structural thicknesses in OI patients and controls.
2. Assess whether OCT/OCTA metrics correlate with cerebral volumetric metrics and visual assessments in OI patients.

## 2 Methods

### 2.1 Participants

This exploratory, observational study was conducted from April 2022 to June 2024 at the West China Hospital of Sichuan University, Chengdu, China. We prospectively recruited unilateral OI patients admitted to the Neurology Department who met the following inclusion criteria: 1. Aged ≥18 years; 2. confirmed OI on MRI, using fluid-attenuated inversion recovery (FLAIR) and diffusion-weighted imaging (DWI); and 3. able to cooperate with the OCT/OCTA examination. The exclusion criteria were as follows: 1. Presence of infarction in other parts of the brain, such as the thalamus, basal ganglia, cerebellum, and brain stem; 2. previous history of stroke; 3. diagnosis of coexisting moderate-to-severe carotid artery stenosis, as determined using computed tomography angiography (CTA) or digital subtraction angiography (DSA); and 4. presence of other neurological disorders, such as Parkinson’s disease or Alzheimer’s disease.

Individuals who denied a history of neurological and ophthalmological disorders, as well as attended our hospital for routine examinations and showed no abnormalities on MRI (Tl-weighted images, T2-weighted images, FLAIR images, and DWI) and OCT/OCTA, were enrolled. Age- and sex-matched individuals were selected for our study as controls.

### 2.2 Data collection

Demographic and medical information, including cardiovascular risk factors such as hypertension, smoking status, diabetes mellitus, hyperlipidemia, and alcohol consumption, was recorded for all participants. All participants underwent a visual acuity examination using a Snellen chart. Each participant’s visual acuity for both eyes was measured under standard lighting conditions and then converted to the logarithm of the minimum angle of resolution (LogMAR).

For OI patients, neurological signs and symptoms were recorded, and the National Institute of Health Stroke Scale (NIHSS) (11) was used to assess the neurological deficit. The disease duration was divided into acute (≤ 14 days) and non-acute (> 14 days) (12). All OI patients underwent echocardiography, electrocardiogram, and CTA to identify the etiology of the stroke.

### 2.3 MR imaging and assessment

**Figure 1:**
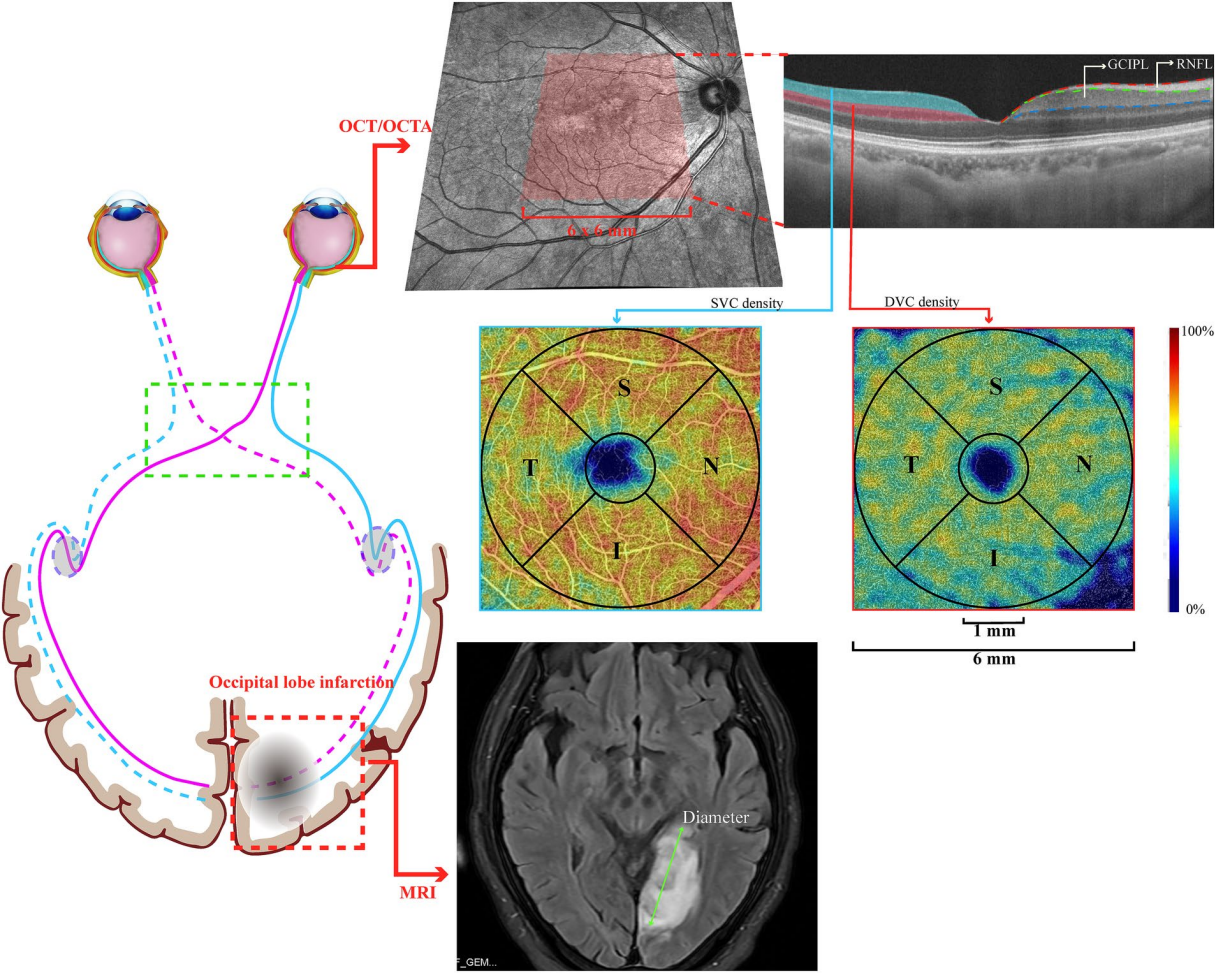
Graphical representation of a patient with occipital lobe infarction, with the OCT/OCTA image.

Our previous reports have fully detailed the brain imaging scanning protocol. Using a 3.0-T MR system (Magnetom Trio, Siemens Medical Systems, Erlangen, Germany), a standardized protocol was used for all patients, including Tl-weighted images, T2-weighted images, FLAIR images, and DWI. The diameter of the infarction was measured on FLAIR images and defined as the maximal diameter of the lesion on the slice with the largest lesion (13) (Figure 1). The eye on the same side as the infarction was defined as the ipsilateral side, while the opposite side was the contralateral side.

### 2.4 Retinal imaging with OCT/OCTA

Swept-source OCT/ OCTA (VG200S; SVision Imaging, Henan, China; version 2.0.106) with eye-tracking technology to reduce motion artifacts was used for retinal imaging in all participants. Our previous reports (14–16) have described the specifications of the OCT/OCTA tool. Structural OCT imaging was conducted with 18 radial B-scans positioned on the fovea. Segmentation of the retinal structural thickness was performed automatically using the OCT tool. Here, we analyzed the macular retinal nerve fiber layer (RNFL) and ganglion cell-inner plexiform layer (GCIPL) within a 6 × 6 mm^2^ area around the fovea, as shown in Figure 1.

OCT angiograms covered an area of 6 × 6 mm^2^ centered on the fovea, produced by the OCTA tool using 512 horizontal B scans. Each B scan contained 512 A scans and was repeated 8 times and averaged. The boundary between the superficial vascular complex (SVC) and deep vascular complex (DVC) was located in the inner two-thirds of the GCIPL, as shown in Figure 1. The mean percentage (%) of the microvasculature in the SVC was obtained using the software in the OCTA tool. OCT and OCTA images with ophthalmic disorders, such as age-macular degeneration, diabetic retinopathy, high myopia, and glaucoma, were excluded. Images with a signal quality of less than 8 or with motion artifacts (presented as horizontal lines) were also excluded from the data analysis. If a participant presented with any of these disorders in one eye, the other eye was used; if both eyes had the aforementioned disorders, the participant was excluded from the study.

In our study, the mean RNFL/GCIPL thicknesses and SVC/DVC densities were measured in the whole area (a circle with a diameter of 6 mm) and in each quadrant [superior (S), temporal (T), nasal (N), and inferior (I)] within an annulus with an outer diameter of 6 mm and an inner diameter of 1 mm, following the Early Treatment Diabetic Retinopathy Study (ETDRS) sectors. The OCT/OCTA data displayed in our study followed the OSCAR-IB quality criteria (17) and APOSTEL recommendation (18).

### 2.5 Statistical analysis

The normality of our data was tested using the Shapiro–Wilk test. Mean ± standard deviation (SD) or median and interquartile ranges (IQRs) were used to describe continuous variables, while frequencies and percentages were used for categorical variables. The *t*-test or Kruskal–Wallis test was used to compare the continuous variables between OI patients and the control group, and Fisher’s exact test was used for the categorical variables. A generalized estimating equation (GEE) was used to compare the OCT/OCTA parameters between OI patients and the controls. A GEE was also used to compare the OCT/OCTA parameters between the controls and OI sub-groups. Multivariable linear regression was used to explore the correlation between the OCT/OCTA parameters and clinic features (infarct diameter and VA) and to compare the OCT/OCTA parameters between the ipsilateral eyes, contralateral eyes, and the control group. Mediation analysis was performed to investigate whether the association between infarct diameter and VA was mediated by the OCT/OCTA parameters. Non-parametric bootstrapping (*B* = 1,000) was used to compute 95% confidence intervals (95% CIs) for the total effect, mediation effects, and direct effects. The covariates for the GEE, linear regression, and mediation analysis included age, sex, hypertension, and inter-eye dependencies. The added variable plot was used to demonstrate the partial correlation between the clinical features and OCT/OCTA parameters. The coordinate axis of the added variable plots represented the residuals of the independent and dependent variables when these variables were regressed on the covariates. Statistical analysis and plotting were conducted using R version 4.2.3 (gee package for GEE, mediation package for mediation analysis). A *p*-value of < 0.05 was considered significant, except for the metrics in the four quadrants, where the Bonferroni method was used, and a p-value of <0.012 was considered significant.

## 3 Results

We identified 71 OI patients; however, we excluded 19 patients because of the presence of infarctions in other parts of the brain and poor retinal imaging quality. Our final data analysis included 52 OI patients (mean age = 56.94 ± 12.66 years; 78.85% male) and 105 controls (mean age = 57.98 ± 9.44 years; 80.95% male). OI patients had a higher burden of hypertension and reduced visual acuity compared to the controls. Of the OI patients, 25 had an infarction in the left hemisphere, while 27 had one in the right hemisphere. In addition, 23 were classified as acute, while 29 were classified as non-acute. OI patients showed lower GCIPL thickness and SVC density (both *p* < 0.001) but higher DVC density (*p* < 0.001) compared to the controls. There was no significant difference in the RNFL thickness between the two groups. Table 1 shows the demographics, clinical data, and OCT/OCTA parameters of our study participants.

**Table 1:** Demographics, clinical features, and OCT/OCTA parameters of the participants.

	Controls	OI	*P*-value
Patients, *n*	105	52	
Eyes, *n*	204	98	
OD	104 (50.98%)	51 (52.04%)	
OS	100 (49.02%)	47 (47.98%)	
Ipsilateral, *n*	-	52 (53.06%)	
Contralateral, *n*	-	46 (46.94%)	
Age, y	57.98 ± 9.44	56.94 ± 12.66	0.564
Sex, male	85 (80.95%)	41 (78.85%)	0.921
Smoking, *n*	28 (26.67%)	25 (48.08%)	0.013
Drinking, *n*	34 (32.38%)	26 (50.00%)	0.05
Diabetes, *n*	11 (10.48%)	15 (28.85%)	0.007
Hypertension, *n*	25 (23.81%)	32 (61.54%)	< 0.001
Dyslipidemia, *n*	11 (10.48%)	4 (7.69%)	0.787
LogVA, LogMAR	−0.02 ± 0.09	−0.23 ± 0.28	< 0.001*
Infraction side			
Left	-	25 (48.07%)	
Right	-	27 (51.93%)	
Stroke etiology	-		
Vertebral artery atherosclerosis		17 (32.69%)	
Basal artery atherosclerosis		2 (3.85%)	
Posterior cerebral artery atherosclerosis		8 (15.34%)	
Vertebral artery dissection		5 (9.62%)	
Cardioembolic		3 (5.77%)	
Cryptogenic		17 (32.69%)	
Infraction phase	-		
Acute		23 (44.23%)	
Non-acute		29 (55.77%)	
RNFL, μm	37.22 ± 3.55	37.86 ± 4.39	0.156*
GCIPL, μm	67.26 ± 5.43	62.3 ± 5.43	< 0.001*
SVC, %	48.09 ± 4.52	44.03 ± 4.58	< 0.001*
DVC, %	51.88 ± 3.50	54.15 ± 3.24	< 0.001*

OCT, optical coherence tomography; OCTA, OCT angiography; OI, occipital lobe infarction; LogMAR, logarithm of the minimum angle of resolution; RNFL, retinal nerve fiber layer; GCIPL, ganglion cell-inner plexiform layer; SVC, superficial vascular complex; DVC, deep vascular complex. ^*^ *p*-value was calculated using a GEE.

**Figure 2:**
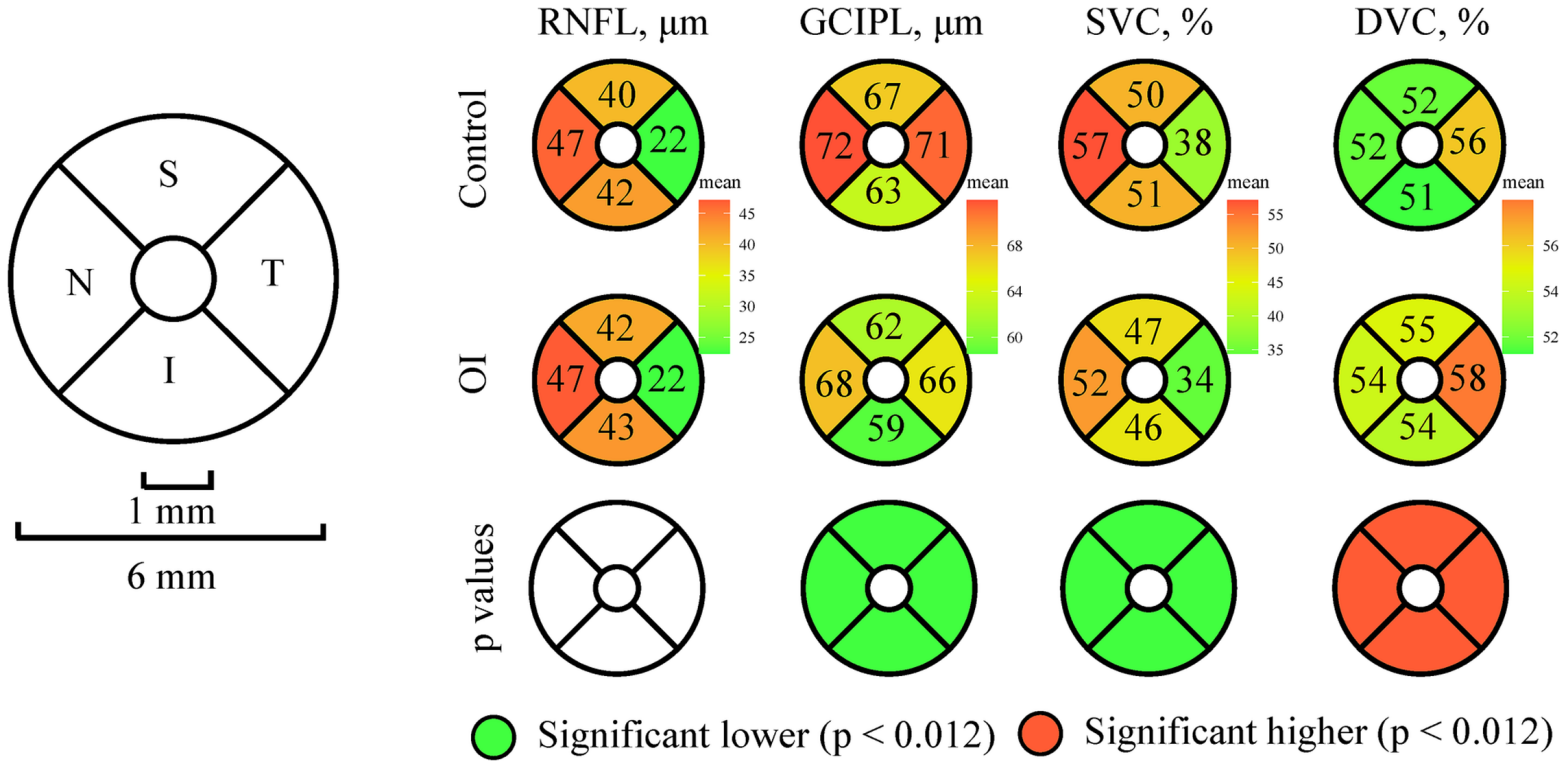
Comparison of the OCT/OCTA parameters across the four sectors between OI patients and controls.

Figure 2 shows the comparison of the OCT/OCTA metrics between OI patients and the controls in the four sectors. OI patients showed lower GCIPL thickness and lower SVC density in all four sectors (all *p* < 0.001) compared to the controls. The DVC density in the four sectors was higher in OI patients than in the controls (all *p* < 0.001). No significant difference was seen in the RNFL thickness across all four sectors when the two groups were compared.

**Figure 3:**
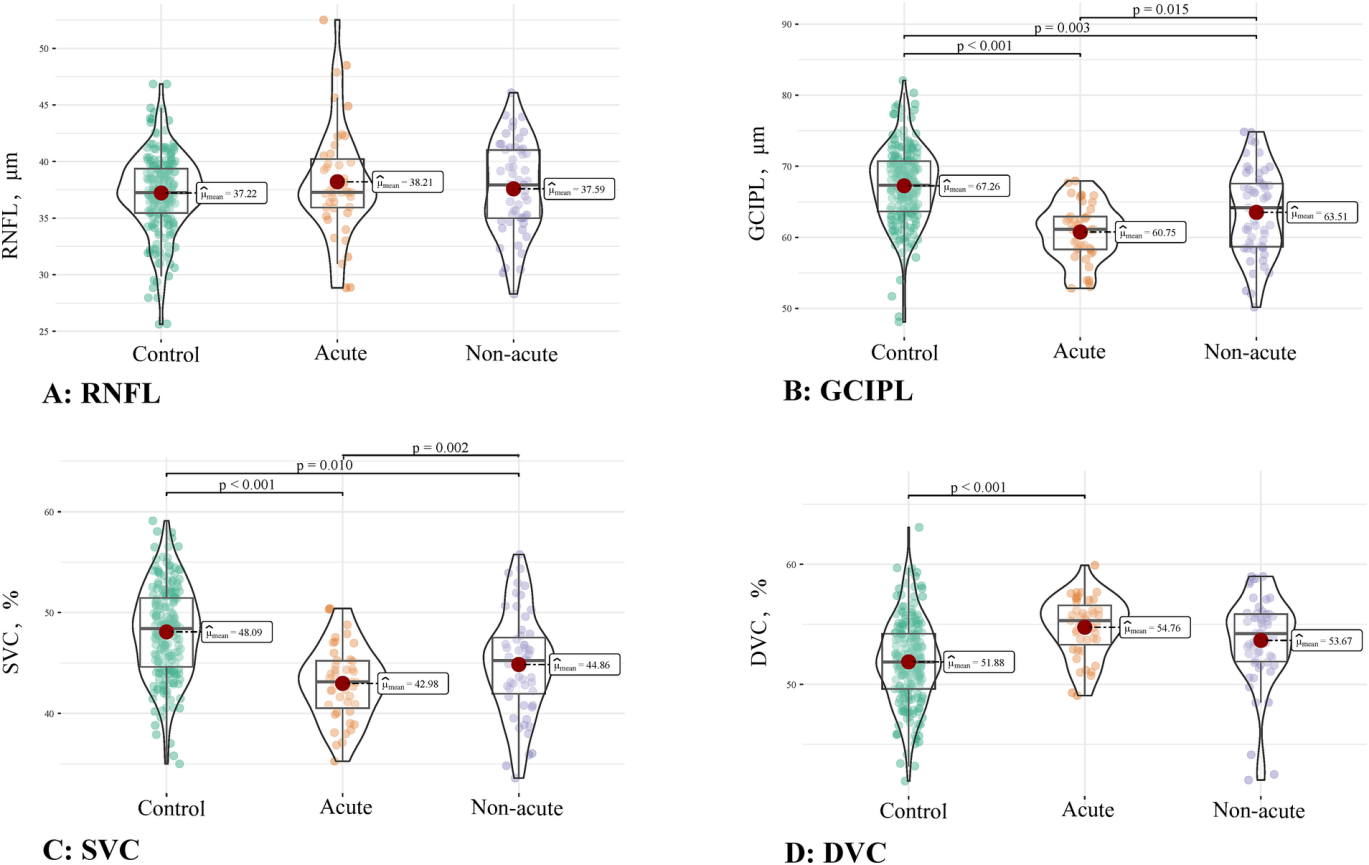
Comparison of the OCT/OCTA parameters among controls, acute OI, and non-acute OI patients. **(A)** RNFL thickness; **(B)** GCIPL thickness; **(C)** SVC density; **(D)** DVC density.

Figure 3 shows the comparison of the OCT/OCTA parameters when OI patients were divided into acute infarction and non-acute infarction groups. Compared to the controls, the patients with acute OI showed reduced GCIPL thickness and lower SVC density (both *p* < 0.001) but higher DVC density (*p* < 0.001). Patients with non-acute OI showed increased GCIPL thickness (*p* = 0.015) and higher SVC density compared to the patients with acute OI (*p* = 0.002). However, compared to the controls, the patients with non-acute OI also showed reduced GCIPL thickness (*p* = 0.003) and lower SVC density (*p* = 0.010). No significant differences in the RNFL thinness were found among the three groups.

Table 2 displays the OCT/OCTA metrics when the eyes of OI patients were stratified into ipsilateral and contralateral to infarction. Compared to the controls, the ipsilateral and contralateral eyes showed reduced GCIPL thicknesses and reduced SVC density (all *p* < 0.05). In addition, the ipsilateral eyes of OI patients showed increased DVC density (*p* < 0.001) compared to the controls. No significant difference (*p* > 0.05) was observed in the RNFL thickness among the controls, ipsilateral eyes, and contralateral eyes. In addition, no significant difference (*p* > 0.05) was observed between the ipsilateral eyes and contralateral eyes for every OCT/OCTA parameter.

**Table 2:** Comparison of the OCT/OCTA parameters among the control eyes and the ipsilateral eyes and contralateral eyes of OI patients.

	Control	Ipsilateral	Contralateral	p1	p2	p3
RNFL, μm	37.22 ± 3.55	38.62 ± 4.19	36.89 ± 4.5	0.384	0.064	0.078
GCIPL, μm	67.26 ± 5.43	62.53 ± 5.47	62.01 ± 5.43	< 0.001	0.004	0.715
SVC, %	48.09 ± 4.52	44.42 ± 4.22	43.54 ± 5.01	< 0.001	0.018	0.521
DVC, %	51.88 ± 3.5	54.22 ± 2.88	54.06 ± 3.69	< 0.001	0.067	0.962

OCT, optical coherence tomography; OCTA, OCT angiography; OI, occipital lobe infarction; RNFL, retinal nerve fiber layer; GCIPL, ganglion cell-inner plexiform layer; SVC, superficial vascular complex; DVC, deep vascular complex. p1 represents the comparison between the control eyes and ipsilateral eyes; p2 represents the comparison between the control eyes and contralateral eyes; and p3 represents the comparison between the ipsilateral eyes and contralateral eyes.

The Supplementary Table 1 shows the comparison of the OCT/OCTA metrics in the four sectors among the control eyes, ipsilateral eyes, and contralateral eyes to infarction. Reduced GCIPL thicknesses and SVC density in all sectors (all *p* < 0.05, except for the temporal sector in the contralateral eyes) were found in the ipsilateral and contralateral eyes compared to the controls.

**Figure 4:**
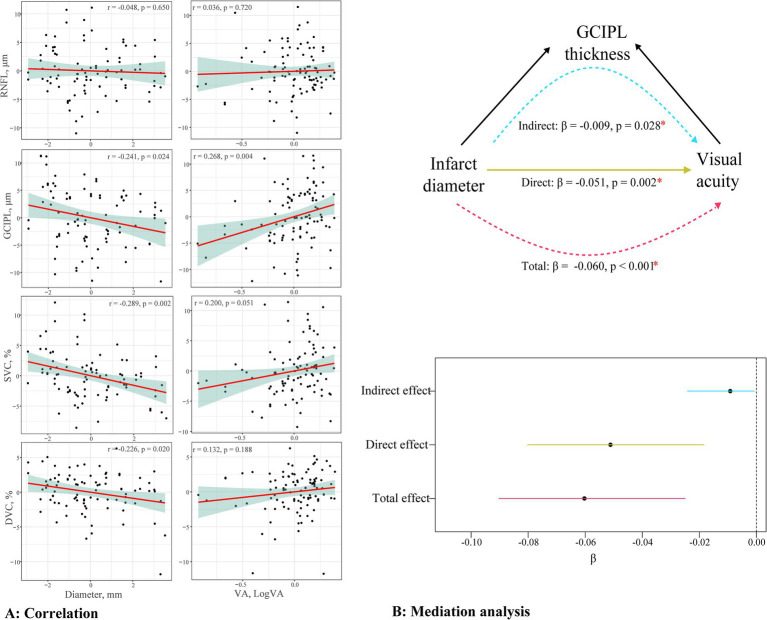
Relationship between the OCT/OCTA parameters and clinical features. **(A)** presents the correlation between the RNFL thickness, GCIPL thickness, SVC density, DVC density, and VA or infract diameter; **(B)** presents the mediation analysis testing the association between the infarct diameter and visual acuity via the thinning of the GCIPL.

Figure 4A shows the association between the OCT/OCTA parameters and clinical features in OI patients. The GCIPL thickness, SVC density, and DVC density significantly correlated with the infarct diameter (all *p* < 0.05). In addition, the GCIPL thickness significantly correlated with visual acuity (*p* = 0.004). No significant correlation (*p* > 0.05) was seen between the RNFL thickness and clinical features in OI patients.

Figure 4B displays the mediation analysis of the association between the infarct diameter and visual acuity via the thinning of the GCIPL in OI patients. The visual acuity showed a significant negative correlation with the infarct diameter (total effect, *β* = −0.060, *p* < 0.001). The thinning of the GCIPL partially mediated this effect (indirect effect, *β* = −0.009, *p* = 0.028), with a proportion of the mediated effect at 15.17%.

## 4 Discussion

In this study, we found that the OCT measures of neuroaxonal loss (GCIPL) were thinner in OI patients compared to controls, which is congruent with previous studies. In addition, patients with acute OI had reduced GCIPL thickness and lower SVC density compared to the controls, while the patients with non-acute OI had increased GCIPL thickness compared to those with acute OI. Moreover, both ipsilateral and contralateral eyes showed reduced GCIPL thicknesses compared to controls, suggesting that neurodegeneration occurs in the bilateral eyes of OI patients. In addition, the GCIPL thickness and SVC density in OI patients were significantly correlated with the infarct diameter, and the visual acuity correlated with the GCIPL thickness. The infarct diameter had a direct effect on visual acuity in OI patients, and the GCIPL thickness partially mediated this effect.

To the best of our knowledge, this is the first report to explore retinal microvascular changes in OI patients. The SVC is responsible for supplying ganglion cells with metabolic energy (19), and thinning of this layer has been observed in previous reports (8, 9, 20). The changes in the SVC observed in our current study further complement the already established OCT structural markers. In addition, previous studies have demonstrated that atherosclerosis is one of the pathological causes of OI, which ultimately leads to ischemia (1, 21). The SVC is believed to reflect cerebral microcirculation and is sensitive to atherosclerosis and ischemic changes in the brain (22, 23). Notably, there is growing evidence that the SVC is also sensitive to cerebral infarction (23, 24). Given that the SVC reflects cerebral microcirculation, we suggest that the lower SVC density in OI patients may indicate a condition (atherosclerosis and/or ischemia) affecting cerebral microcirculation. In contrast, OI patients showed higher DVC density compared to controls. Future studies are needed to further elucidate this finding.

Previous studies (3, 8, 25) have demonstrated that transsynaptic retrograde neurodegeneration after OI is aggressive during the acute phase and stable over the years. In this study, we showed that acute OI patients had reduced GCIPL thickness and lower SVC density compared to the controls. In addition, the GCIPL thickness in acute OI patients was reduced compared to non-acute OI patients. These findings suggest that retinal neurodegeneration and microvascular impairment follow a clear-cut time sequence where retrograde degeneration and microvascular impairment are progressive and steady over time. The finding that the acute infarction group had thinner GCIPL than the non-acute group in the study may partly be attributed to inevitable selective bias. Patients with obvious visual symptoms tend to visit the emergency department, while those without visual symptoms (usually complaining of dizziness) tend to visit outpatient services. As a result, the former patients are included in the acute group and may have experienced more severe visual path damage and degeneration.

Clinically, the infarct lesion size reflects neurological damage. Here, we showed that the GCIPL thickness in OI patients is inversely correlated with the infarct diameter. GCIPL thickness is mainly composed of retinal ganglion cells (26) and is believed to reflect neuronal damage in stroke (27). We suggest that a larger infarct diameter is associated with more severe thinning of the GCIPL in OI patients; thus, GCIPL integrity in OI patients may reflect the lesion range.

Accumulating reports (2, 6) have shown that OI is linked with visual impairment. In this study, we showed that reduced visual acuity in OI patients correlated with the thinning of the GCIPL. The GCIPL is composed of the cell bodies and dendrites of retinal ganglion cells, which play a significant role in visual processing (28). The association between GCIPL thickness and VA suggests that neurodegeneration in this retinal layer in OI patients may be associated with VA. Our finding highlights the importance of preventing early neurodegeneration, particularly ganglion cell death, to preserve vision and prevent further deterioration. This finding could be useful to other researchers exploring therapeutic targets and developing early prevention strategies for OI in the future.

It is of great interest to investigate retinal changes in hemifields due to hemianopsia. It is expected that retinal thinning would be observed in the temporal half of the ipsilateral eyes to occipital lobe infarction and in the nasal half of the contralateral eyes. However, this expected result was not found in our cohort; our study showed GCIPL thinning and reduced SVC density in all sectors of both ipsilateral and contralateral eyes. This may be due to the limited sample size, and not all patients exhibited hemianopsia. Further studies should consider combining OCT/OCTA examination with visual field testing.

Infarction in the occipital lobe has several deleterious effects on vision. Based on previous research, we identified the thinning of the retinal sub-layers (macular RNFL and GCIPL) as likely mediators of visual loss and/or impairment in OI patients. We found partial mediation between the GCIPL thinning and infarct diameter in OI patients, suggesting that thinning of the GCIPL is an important pathological process disrupting VA in OI patients. Damage to brain regions involved in visual processing can lead to damage to or disruption of connections within the visual tract, causing retrograde degeneration in the retina (2, 7). Visual complaints and symptoms have repeatedly been reported in OI patients, implicating structural changes in the retina. We also showed that the infarct diameter in OI patients directly resulted in reduced VA. Damage to brain regions involved in vision results in visual loss and/or visual impairment, making it reasonable to suggest that OI directly affects vision.

Several limitations of the study must be acknowledged. First, our sample size was small due to the strict selection criteria; we excluded OI patients with infarctions in other areas of the brain, such as the thalamus, hippocampus, cerebellum, and brain stem. The study’s cross-sectional design limits the ability to interpret the findings as cause and effect, although mediation analysis was conducted. The description of retinal changes and disease period was also limited. Our data cannot be generalized to the entire population because the results were derived from a single center. In addition, participants who could not undergo OCT/OCTA imaging due to ocular comorbidities were excluded, potentially introducing selection bias. Visual field testing was not conducted in our current study, which limited the exploration of the relationship between retinal changes and visual function in OI. Further studies should consider combining OCT/OCTA examination with visual field testing. The strengths of this study include the quantification of retinal sub-layer thicknesses and microvasculature and the measurement of the infarct diameter in OI patients.

## 5 Conclusion

In conclusion, OI patients had reduced retinal sub-layer thicknesses and lower retinal microvascular density compared to controls. The thinner GCIPL was associated with reduced visual acuity and a larger infarct diameter, while the lower SVC density correlated with a larger infarct diameter. Furthermore, a larger infarct diameter in OI patients directly affected the visual acuity, and the GCIPL thickness partially mediated this effect. Retinal structural thickness measured using OCT has the potential to detect neurodegeneration in occipital lobe infarction.

## Data Availability

The raw data supporting the conclusions of this article will be made available by the authors, without undue reservation.
