# Supplementary material for: Retinal thickness is indicative of visual loss in patients with occipital lobe infarction
Source: Front Neurol. 2025 Mar 25;16:1546439. doi: 10.3389/fneur.2025.1546439 (PMC11975593; doi:10.3389/fneur.2025.1546439)
Supplement: Supplementary file 1 [file Table_1.docx]

Supplementary Table

Comparison of OCT/OCTA parameters in four sectors among control eyes, ipsilateral eyes, and contralateral eyes of OI patients.

|  | Control | Ipsilateral | Contralateral | p1 | p2 | p3 |
| --- | --- | --- | --- | --- | --- | --- |
| RNFL, μm | | | | | | |
| Superior | 40.21±4.58 | 42.22±5.15 | 40.78±5.41 | 0.047 | 0.070 | 0.216 |
| Temporal | 22.37±2.89 | 22.86±3.27 | 21.9±2.54 | 0.946 | 0.216 | 0.153 |
| Inferior | 42.48±4.84 | 43.42±5.22 | 42.4±6.79 | 0.475 | 0.163 | 0.474 |
| Nasal | 46.56±5.87 | 48.43±7.44 | 45.37±6.55 | 0.935 | 0.108 | 0.057 |
| GCIPL, μm | | | | | | |
| Superior | 67.07±6.05 | 61.98±6.07 | 61.7±5.74 | <0.001 | 0.014 | 0.899 |
| Temporal | 71.25±6.53 | 65.83±7.07 | 65.55±6.33 | <0.001 | 0.006 | 0.886 |
| Inferior | 63.17±5.34 | 58.88±5.92 | 58.19±5.66 | <0.001 | 0.007 | 0.616 |
| Nasal | 71.93±6.03 | 67.91±5.2 | 67.17±6.47 | <0.001 | 0.020 | 0.604 |
| SVC, % | | | | | | |
| Superior | 50.43±5.72 | 47.51±5.3 | 46.05±6.52 | <0.001 | 0.034 | 0.307 |
| Temporal | 38.16±4.18 | 34.2±3.64 | 34.78±4.1 | <0.001 | 0.121 | 0.300 |
| Inferior | 50.62±5.26 | 46.39±5.7 | 45.83±6.57 | <0.001 | 0.043 | 0.778 |
| Nasal | 57.06±6.12 | 53.11±6.12 | 51.45±6.57 | <0.001 | 0.022 | 0.33 |
| DVC, % | | | | | | |
| Superior | 51.81±4.52 | 55.41±3.5 | 53.86±6.23 | <0.001 | 0.121 | 0.177 |
| Temporal | 56.2±3.55 | 57.83±4.34 | 58.18±3.69 | <0.001 | 0.493 | 0.466 |
| Inferior | 51.27±4.62 | 53.96±3.57 | 53.2±5.88 | <0.001 | 0.055 | 0.534 |
| Nasal | 51.74±4.84 | 53.71±4.57 | 54.96±4.53 | <0.001 | 0.242 | 0.144 |

OCT: optical coherence tomography; OCTA: OCT angiography; OI: occipital lobe infarction; RNFL: retinal nerve fiber layer; GCIPL: ganglion cell-inner plexiform layer; SVC: superficial vascular complex; DVC: deep vascular complex. p1 represents comparison between control eyes and ipsilateral eyes; p2 represents comparison between control eyes and contralateral eyes; p3 represent comparison between ipsilateral eyes and contralateral eyes
